# 2-[(Isopropoxycarbonothio­yl)sulfanyl]­acetic acid

**DOI:** 10.1107/S1600536810041267

**Published:** 2010-11-06

**Authors:** Shude Xiao, Paul A. Charpentier

**Affiliations:** a Deptartment of Chemical and Biochemical Engineering, Faculty of Engineering, The University of Western Ontario, London, Ontario, Canada N6A 5B9

## Abstract

The title compound, C_6_H_10_O_3_S_2_, features a planar C atom connected to one O and two S atoms, the C—S single bond being distinctly longer than the C–S double bond. Two mol­ecules are linked by an O—H⋯O hydrogen bond about a center of inversion, generating a dimer.

## Related literature

For general background to the synthesis and applications of the title compound, see: Stenzel *et al.* (2003 ▶); Moad *et al.* (2005 ▶, 2008 ▶). For applications in polymerization, see: Coote & Radom (2004 ▶); Favier *et al.* (2004 ▶).
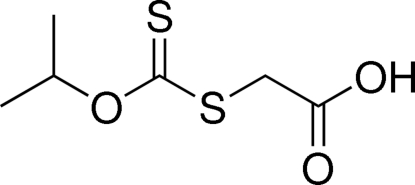


## Experimental

### 

#### Crystal data

C_6_H_10_O_3_S_2_
*M* *_r_* = 194.26
Monoclinic, 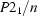

*a* = 5.0092 (14) Å
*b* = 7.712 (2) Å
*c* = 23.868 (7) Å
β = 90.294 (9)°
*V* = 922.0 (4) Å^3^
*Z* = 4
Mo *K*α radiation
μ = 0.54 mm^−1^
*T* = 150 K
0.05 × 0.02 × 0.02 mm

#### Data collection

Bruker APEXII CCD diffractometer
Absorption correction: multi-scan (*SADABS*; Bruker, 2009 ▶) *T*_min_ = 0.972, *T*_max_ = 0.992
6469 measured reflections
2040 independent reflections
1306 reflections with *I* > 2σ(*I*)
*R*_int_ = 0.061

#### Refinement

*R*[*F* ^2^ > 2σ(*F* ^2^)] = 0.046
*wR*(*F* ^2^) = 0.095
*S* = 1.03
2040 reflections
103 parameters
H-atom parameters constrained
Δρ_max_ = 0.34 e Å^−3^
Δρ_min_ = −0.30 e Å^−3^

### 

Data collection: *APEX2* (Bruker, 2009 ▶); cell refinement: *SAINT* (Bruker, 2009 ▶); data reduction: *SAINT*; program(s) used to solve structure: *SHELXS97* (Sheldrick, 2008 ▶); program(s) used to refine structure: *SHELXL97* (Sheldrick, 2008 ▶); molecular graphics: *SHELXTL* (Sheldrick, 2008 ▶); software used to prepare material for publication: *SHELXTL*.

## Supplementary Material

Crystal structure: contains datablocks global, I. DOI: 10.1107/S1600536810041267/ng5039sup1.cif

Structure factors: contains datablocks I. DOI: 10.1107/S1600536810041267/ng5039Isup2.hkl

Additional supplementary materials:  crystallographic information; 3D view; checkCIF report

## Figures and Tables

**Table 1:** Hydrogen-bond geometry (Å, °)

*D*—H⋯*A*	*D*—H	H⋯*A*	*D*⋯*A*	*D*—H⋯*A*
O2—H2⋯O3^i^	0.84	1.83	2.664 (3)	174

Symmetry code: (i) 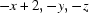.

**Fig. 1.**
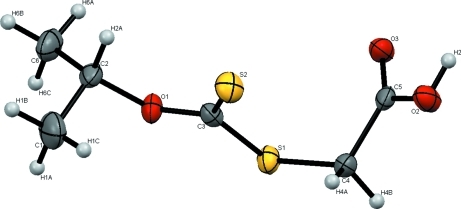
View of the title compound (50% probability displacement ellipsoids).

**Fig. 2.**
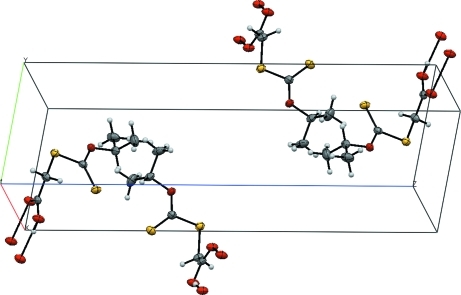
Packing diagram of the structure with H-bonds.

## supplementary crystallographic information

### Comment

In reversible addition-fragmentation chain-transfer (RAFT) polymerization, xanthates are used as chain transfer agents (CTA) for reversible-deactivation radical polymerization (RDRP) of vinyl acetate (Moad *et al.*, 2005, 2008). Vinyl acetate is one of the typical monomers that cannot be easily polymerized in RDRP, because vinyl acetate radicals are highly unstable. However, xanthates destabilize the intermediate radicals in the RAFT equilibriums, and RDRP can be achieved (Coote & Radom, 2004; Favier *et al.*, 2004). Stenzel *et al.* (2003) synthesized 2-(isopropoxycarbonothioylthio)acetate as the CTA to mediate the polymerization of vinyl acetate, but lack of functionality limits its applications. Therefore, 2-(isopropoxycarbonothioylthio)acetic acid was synthesized. It was employed in RAFT polymerization of vinyl acetate, with poly(vinyl acetate) having carboxylic acid end groups successfully obtained.

Investigation of the single-crystal of 2-(isopropoxycarbonothioylthio)acetic acid was conducted to understand its structural properties.

### Experimental

Potassium hydroxide 5.6 g (50 mmol) and 2-propanol 100 ml were mixed to form a homogeneous solution, after which carbon disulfide 20 ml was added dropwise at room temperature. The mixture was kept stirred for 1 day at 40 °C. Then the solvent and residual carbon disulfide were evaporated to obtrain a light yellow powder. The powder was dissolved in methanol, and mixed with the methanol solution of bromoacetic acid. The reaction was conducted at 40 °C for 20 h. Salts were filtered out and solvents were evaporated. The oil was washed with excess diluted hydrochloric acid and extracted with ethyl ether. The crude product was run through a silica gel column with a solvent mixture of ethyl ether/hexanes (1:2). Colorless crystals of 2-(isopropoxycarbonothioylthio)acetic acid were obtained from recrystalization in hexanes. m.p. 44.3°C (DSC). MS: 194.0078.

### Refinement

The hydrogen atom positions were calculated geometrically and were included as riding on their respective carbon/oxygen atoms.

### Crystal data

**Table:**

C_6_H_10_O_3_S_2_	*F*(000) = 408
*M**_r_* = 194.26	*D*_x_ = 1.399 Mg m^−^^3^
Monoclinic, *P*2_1_/*n*	Mo *K*α radiation, λ = 0.71073 Å
Hall symbol: -P 2yn	Cell parameters from 981 reflections
*a* = 5.0092 (14) Å	θ = 2.8–23.5°
*b* = 7.712 (2) Å	µ = 0.54 mm^−^^1^
*c* = 23.868 (7) Å	*T* = 150 K
β = 90.294 (9)°	Block, colourless
*V* = 922.0 (4) Å^3^	0.05 × 0.02 × 0.02 mm
*Z* = 4	

### Data collection

**Table:**

Bruker APEXII CCD diffractometer	2040 independent reflections
Radiation source: fine-focus sealed tube	1306 reflections with *I* > 2σ(*I*)
graphite	*R*_int_ = 0.061
φ and ω scans	θ_max_ = 27.1°, θ_min_ = 1.7°
Absorption correction: multi-scan (*SADABS*; Bruker, 2009)	*h* = −3→6
*T*_min_ = 0.972, *T*_max_ = 0.992	*k* = −9→9
6469 measured reflections	*l* = −30→30

### Refinement

**Table:**

Refinement on *F*^2^	Primary atom site location: structure-invariant direct methods
Least-squares matrix: full	Secondary atom site location: difference Fourier map
*R*[*F*^2^ > 2σ(*F*^2^)] = 0.046	Hydrogen site location: inferred from neighbouring sites
*wR*(*F*^2^) = 0.095	H-atom parameters constrained
*S* = 1.03	*w* = 1/[σ^2^(*F*_o_^2^) + (0.0365*P*)^2^ + 0.0157*P*] where *P* = (*F*_o_^2^ + 2*F*_c_^2^)/3
2040 reflections	(Δ/σ)_max_ < 0.001
103 parameters	Δρ_max_ = 0.34 e Å^−^^3^
0 restraints	Δρ_min_ = −0.30 e Å^−^^3^

### Special details

**Table:**

Geometry. All e.s.d.'s (except the e.s.d. in the dihedral angle between two l.s. planes) are estimated using the full covariance matrix. The cell e.s.d.'s are taken into account individually in the estimation of e.s.d.'s in distances, angles and torsion angles; correlations between e.s.d.'s in cell parameters are only used when they are defined by crystal symmetry. An approximate (isotropic) treatment of cell e.s.d.'s is used for estimating e.s.d.'s involving l.s. planes.
Refinement. Refinement of *F*^2^ against ALL reflections. The weighted *R*-factor *wR* and goodness of fit *S* are based on *F*^2^, conventional *R*-factors *R* are based on *F*, with *F* set to zero for negative *F*^2^. The threshold expression of *F*^2^ > σ(*F*^2^) is used only for calculating *R*-factors(gt) *etc*. and is not relevant to the choice of reflections for refinement. *R*-factors based on *F*^2^ are statistically about twice as large as those based on *F*, and *R*- factors based on ALL data will be even larger.

### Fractional atomic coordinates and isotropic or equivalent isotropic displacement parameters (Å^2^)

**Table:**

	*x*	*y*	*z*	*U*_iso_*/*U*_eq_	
S1	0.62574 (14)	0.49105 (9)	0.06736 (3)	0.0265 (2)	
S2	0.97062 (15)	0.32368 (10)	0.15640 (3)	0.0302 (2)	
O1	0.9373 (4)	0.6573 (2)	0.12779 (8)	0.0252 (5)	
O2	0.7271 (4)	−0.0108 (2)	0.04984 (9)	0.0311 (5)	
H2	0.8469	−0.0692	0.0341	0.047*	
O3	0.9230 (4)	0.2085 (2)	0.00436 (8)	0.0288 (5)	
C1	0.9765 (7)	0.7333 (4)	0.22585 (13)	0.0456 (9)	
H1A	0.8459	0.8265	0.2205	0.068*	
H1B	1.1010	0.7651	0.2559	0.068*	
H1C	0.8835	0.6260	0.2359	0.068*	
C2	1.1292 (5)	0.7057 (4)	0.17223 (12)	0.0267 (7)	
H2A	1.2634	0.6110	0.1775	0.032*	
C3	0.8641 (5)	0.4925 (3)	0.12137 (11)	0.0223 (6)	
C4	0.5442 (5)	0.2657 (3)	0.06314 (12)	0.0246 (7)	
H4A	0.5113	0.2220	0.1015	0.030*	
H4B	0.3758	0.2532	0.0416	0.030*	
C5	0.7540 (5)	0.1537 (4)	0.03631 (11)	0.0226 (6)	
C6	1.2649 (6)	0.8665 (4)	0.15058 (15)	0.0408 (9)	
H6A	1.3543	0.8400	0.1152	0.061*	
H6B	1.3971	0.9064	0.1781	0.061*	
H6C	1.1315	0.9576	0.1444	0.061*	

### Atomic displacement parameters (Å^2^)

**Table:**

	*U*^11^	*U*^22^	*U*^33^	*U*^12^	*U*^13^	*U*^23^
S1	0.0297 (4)	0.0200 (4)	0.0297 (4)	0.0018 (3)	−0.0059 (3)	−0.0032 (4)
S2	0.0362 (4)	0.0214 (4)	0.0330 (4)	0.0034 (3)	−0.0032 (3)	0.0049 (4)
O1	0.0320 (11)	0.0181 (11)	0.0255 (11)	−0.0002 (8)	−0.0079 (9)	−0.0033 (9)
O2	0.0313 (11)	0.0196 (11)	0.0426 (13)	−0.0001 (9)	0.0099 (10)	−0.0015 (11)
O3	0.0268 (11)	0.0223 (11)	0.0375 (12)	−0.0038 (9)	0.0091 (10)	−0.0040 (10)
C1	0.052 (2)	0.052 (2)	0.0323 (19)	−0.0011 (18)	−0.0065 (18)	−0.0151 (18)
C2	0.0212 (14)	0.0253 (16)	0.0334 (17)	0.0003 (13)	−0.0074 (13)	−0.0061 (15)
C3	0.0252 (14)	0.0200 (14)	0.0218 (15)	0.0024 (13)	0.0043 (12)	−0.0025 (14)
C4	0.0203 (15)	0.0236 (16)	0.0299 (16)	−0.0029 (12)	0.0013 (13)	−0.0061 (13)
C5	0.0201 (14)	0.0205 (16)	0.0270 (16)	−0.0027 (12)	−0.0053 (13)	−0.0052 (14)
C6	0.0362 (18)	0.0232 (17)	0.063 (2)	−0.0052 (14)	−0.0075 (17)	−0.0075 (18)

### Geometric parameters (Å, °)

**Table:**

S1—C3	1.753 (3)	C1—H1B	0.9800
S1—C4	1.788 (3)	C1—H1C	0.9800
S2—C3	1.635 (3)	C2—C6	1.506 (4)
O1—C3	1.331 (3)	C2—H2A	1.0000
O1—C2	1.476 (3)	C4—C5	1.506 (4)
O2—C5	1.316 (3)	C4—H4A	0.9900
O2—H2	0.8400	C4—H4B	0.9900
O3—C5	1.218 (3)	C6—H6A	0.9800
C1—C2	1.509 (4)	C6—H6B	0.9800
C1—H1A	0.9800	C6—H6C	0.9800
			
C3—S1—C4	101.66 (13)	S2—C3—S1	126.18 (17)
C3—O1—C2	120.2 (2)	C5—C4—S1	114.94 (19)
C5—O2—H2	109.5	C5—C4—H4A	108.5
C2—C1—H1A	109.5	S1—C4—H4A	108.5
C2—C1—H1B	109.5	C5—C4—H4B	108.5
H1A—C1—H1B	109.5	S1—C4—H4B	108.5
C2—C1—H1C	109.5	H4A—C4—H4B	107.5
H1A—C1—H1C	109.5	O3—C5—O2	124.1 (3)
H1B—C1—H1C	109.5	O3—C5—C4	123.8 (3)
O1—C2—C6	104.8 (2)	O2—C5—C4	112.1 (2)
O1—C2—C1	108.3 (2)	C2—C6—H6A	109.5
C6—C2—C1	114.0 (3)	C2—C6—H6B	109.5
O1—C2—H2A	109.9	H6A—C6—H6B	109.5
C6—C2—H2A	109.9	C2—C6—H6C	109.5
C1—C2—H2A	109.9	H6A—C6—H6C	109.5
O1—C3—S2	127.8 (2)	H6B—C6—H6C	109.5
O1—C3—S1	106.06 (19)		

### Hydrogen-bond geometry (Å, °)

**Table:**

*D*—H···*A*	*D*—H	H···*A*	*D*···*A*	*D*—H···*A*
O2—H2···O3^i^	0.84	1.83	2.664 (3)	174

Symmetry codes: (i) −*x*+2, −*y*, −*z*.
